# Impact of gender on tumor stage and survival of upper urinary tract urothelial cancer: A population-based study

**DOI:** 10.1007/s00508-016-1088-4

**Published:** 2016-09-26

**Authors:** Badereddin Mohamad Al-Ali, Stephan Madersbacher, Nadine Zielonke, Ingrid Schauer, Thomas Waldhoer, Gerald Haidinger

**Affiliations:** 1 Department of Urology, Kaiser-Franz-Josef-Spital, Vienna, Austria; 2 Austrian National Cancer Registry, Statistics Austria, Vienna, Austria; 3 Department of Epidemiology, Centre for Public Health, Medical University of Vienna, Kinderspitalgasse 15, 1090 Vienna, Austria; 4 Department of Urology, Medical University of Graz, Graz, Austria

**Keywords:** Urothelial carcinoma, Upper urinary tract, Sex, Radical nephroureterectomy, Tumor stage

## Abstract

**Background:**

The aim of this study was to analyze the impact of gender on tumor stage, overall and cancer-specific mortality of upper urinary tract urothelial cancer (UTUC) in a population-based, nationwide analysis.

**Methods:**

All Austrian patients with UTUC diagnosed between 1983 and 2010 were included in this study. Overall mortality was estimated by the Kaplan-Meier method. Cancer-specific (UTUC) mortality was estimated by cumulative incidence with mortality due to other causes as a competing risk. The effect of age was adjusted in a descriptive as well as a statistical inferential way.

**Results:**

This study included 2066 patients (men *n* = 1169, mean age 68.3 ±11.5 years, women *n* = 897, 72.6 ±10.4 years). Tumor stage distribution was as follows: pT1: men *n* = 411, women *n* = 268, pT2: men *n* = 263, women *n* = 187, pT3: men *n* = 382, women *n* = 328 and pT4: men *n* = 113, women *n* = 114. The male:female ratio continuously declined from 1.5 for pT1 tumors to 1.4 for pT2 tumors, 1.2 for pT3 tumors and 1.0 for pT4-tumors. In the entire cohort the 5‑year cumulative overall mortality was 57 % for women versus 50 % for men (*p* = 0.0002). For pT1 (women 33 %, men 31 %) and pT2 stage tumors (women 45 %, men 45 %) the 5‑year overall mortality was comparable between both sexes. In pT3 (women 68 %, men 62 %) and pT4 (women 95 %, men 87 %) tumors women had a higher overall mortality rate. The 5‑year cancer-specific mortality (CSM) of the entire cohort was 12 % for women and 10 % for men (*p* = 0.067): pT1 women 5 % men 3 %, pT2 women 9 % men 10 %, pT3 women 14 % men 11 % and pT4 women 29 % men 27 %.

**Conclusions:**

In this population-based nationwide analysis, sex differences were notable for UTUC. Women tended to have more advanced tumor stages at diagnosis and a higher overall and cancer-specific mortality in advanced tumor stages.

## Introduction

Upper urinary tract urothelial carcinoma (UTUC) is a rare urological malignancy accounting for approximately 5–6 % of all urothelial tumors with an incidence of 0.7 per 100,000 person-years [1]. Despite the increased detection of earlier stage tumors resulting from the recent improvements in imaging and endoscopic techniques, UTUC remains an aggressive disease with high recurrence and progression rates [1, 2].

Prognostic predictors of UTUC can be grossly divided into clinical, demographic and pathological factors. Demographic factors involve age, obesity, performance status, smoking and race [1]. Pathological factors include tumor stage, grade, concomitant carcinoma in situ, lymph node invasion, tumor architecture and lymphovascular invasion [1, 2].

The impact of gender on the incidence and prognosis of patients with UTUC is still poorly investigated. Comparable to the situation for bladder cancer, the role of gender for UTUC is intensively discussed. The occurrence of UTUC is 2–3 times more common in men and sex differences relating to clinical and pathological characteristics have been reported but with conflicting findings [3, 4]. The vast majority of previous studies on this topic are hampered by relatively small sample sizes and/or potential selection bias of data reported by tertiary referral centers.

We therefore aimed to assess the impact of sex on (i) tumor stage of UTUC at diagnosis and (ii) on overall and cancer specific mortality in a population-based, nationwide analysis. To this end we linked the national cancer registry to the national death statistics. For decades, Austria (total population 8.4 million) has had an equal access healthcare system with compulsory state insurance and a national cancer registry.

## Material and methods

Data on cancer incidence were obtained from the Austrian National Cancer Registry. All patients with UTUC with tumor stages pT1, pT2, pT3 and pT4 diagnosed between 1983 until 2010 were followed up. The information collected included the following variables: date of birth, sex, date of diagnosis, stage, site and histological type of the tumor according to the Standard International Classification of Diseases for Oncology (ICD-O-3).

The cancer incidence database is regularly matched with official death certificates by Statistics Austria. Statistics related to the causes of death (Statistics Austria) were used for passive follow-up and information related to the date of death and cause of death was obtained. Complete data were recoded into ICD-10 and included incidence as well as survival data of codes C65 and C66 (malignant neoplasm of renal pelvis and malignant neoplasm of ureter). The overall mortality was estimated by the Kaplan-Meier method, UTUC-specific mortality was estimated by cumulative incidence using the %CIF macro provided by SAS (SAS Institute, Cary, NC) with mortality due to other causes as a competing risk. The SAS macro allows estimation of cumulative incidence functions with competing risks and provides a comparison of cumulative incidence functions by the Gray test [5]. Furthermore, the effect of sex was adjusted by age at diagnosis by means of a Cox as well as a Fine-Gray regression model using the phreg in SAS. For descriptive purposes only, age at diagnosis was grouped into <70 years and >70 years and cumulative incidence was calculated for each group. Corresponding *p*-values are explorative in the sense that no prespecified hypotheses were set up in advance of the study and no adjustments for multiple tests were applied. Median follow-up times were calculated by the reverse Kaplan-Meier method.

Tumors were staged according to the 2002 American Joint Committee on Cancer and International Union against Cancer (AJCC/UICC) TNM classification [6]. Tumor grade was assessed according to the 1998 World Health Organization and International Society of Urological Pathology consensus classification [7].

## Results

### Patient characteristics

Between 1983 and 2010 a total of 2066 patients including 1169 men (56.6 %) and 897 women (43.4 %) with UTUC were identified and included in this study (Table 1). The median follow-up of the male cohort was 13.4 years (95 % confidence interval CI 12.3–14.9 years) and of the female cohort 14.5 years (95%CI 13.2–15.9 years). The mean age of the entire cohort was 70.1 ± 11.2 years (women 72.6 ± 10.4 years, men 68.3 ± 11.4 years, *p* = 0.0017). This sex-specific age difference at diagnosis was demonstrable for all tumor stages (Table 1). The tumor stage distribution within sex was as follows: pT1 men *n* = 411 (35.2 %), women *n* = 268 (28.9 %), pT2 men *n* = 263 (22.5 %), women *n* = 187 (20.8 %), pT3 men *n* = 382 (32.7 %), women *n* = 328 (36.6 %) and pT4 men *n* = 113 (9.6 %), women *n* = 114 (12.7 %). The male: female ratio continuously declined from 1.5 for pT1 tumors to 1.4 for pT2 tumors, 1.2 for pT3 tumors and 1.0 for pT4 tumors.

**Table 1:** Age at diagnosis and median follow-up per period of patients diagnosed with upper urinary tract urothelial cancer according to gender and tumor stage

Overall	Number (*n*)	Age (years, mean ± SD)	Follow-up median estimated value in years (95 % CI)
Women total	897	72.6 ± 10.4	14.5 (13.2–15.8)
Men total	1169	68.3 ± 11.5	13.4 (12.3–14.9)
pT1 female	268	72 ± 11.6	15.4 (13.9–17.1)
pT2 female	187	71.7 ± 10.7	15.6 (12.5–20.8)
pT3 female	328	73.4 ± 9.4	11.6 (10.2–14.6)
pT4 female	114	73.0 ± 9.7	13.7 (3.2–22.5)
pT1 male	411	66.7 ± 11.6	12.9 (11.6–15.5)
pT2 male	263	68.6 ± 11.7	16.2 (14.7–18.9)
pT3 male	382	69.1 ± 11.1	11.3 (10.3–14.4)
pT4 male	113	70.4 ± 11.3	11.4 (3.6–13.5)

*pT1–pT4* tumor stage according to the AJCC/UICC TEM classification

In the male cohort there was an association between age and tumor stage, i. e. advanced stages in higher age groups: pT1 66.7 ± 11.6 years (mean ± SD), pT2 68.6 ± 11.7 years, pT3 69.2 ± 11.1 years and pT4 70.4 ± 11.3 years. This age trend, however, was not present in women: pT1 72.0 ± 11.6 years, pT2 71.7 ± 10.7 years, pT3 73.3 ± 9.4 years and pT4 73.0 ± 9.8 years.

### Cumulative overall mortality

**Fig. 1:**
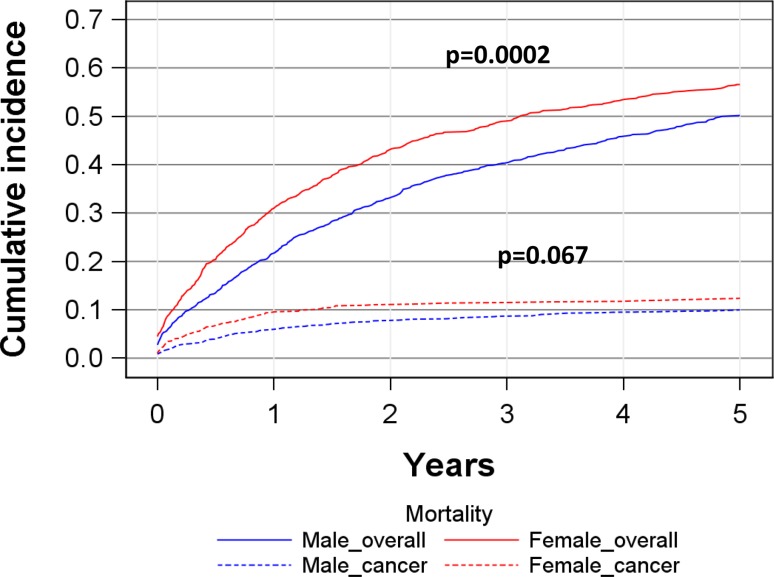
Cumulative overall and cancer specific mortality in total study cohort

**Fig. 2:**
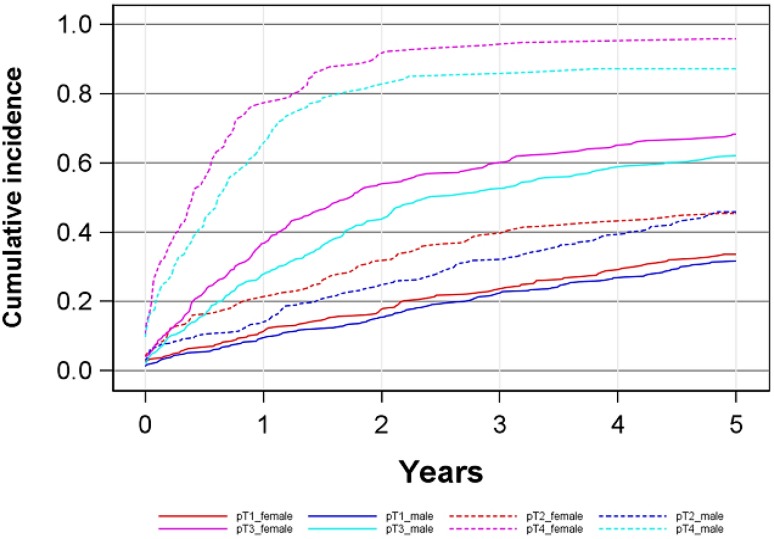
Cumulative overall mortality depending on sex and tumor stage

**Fig. 3:**
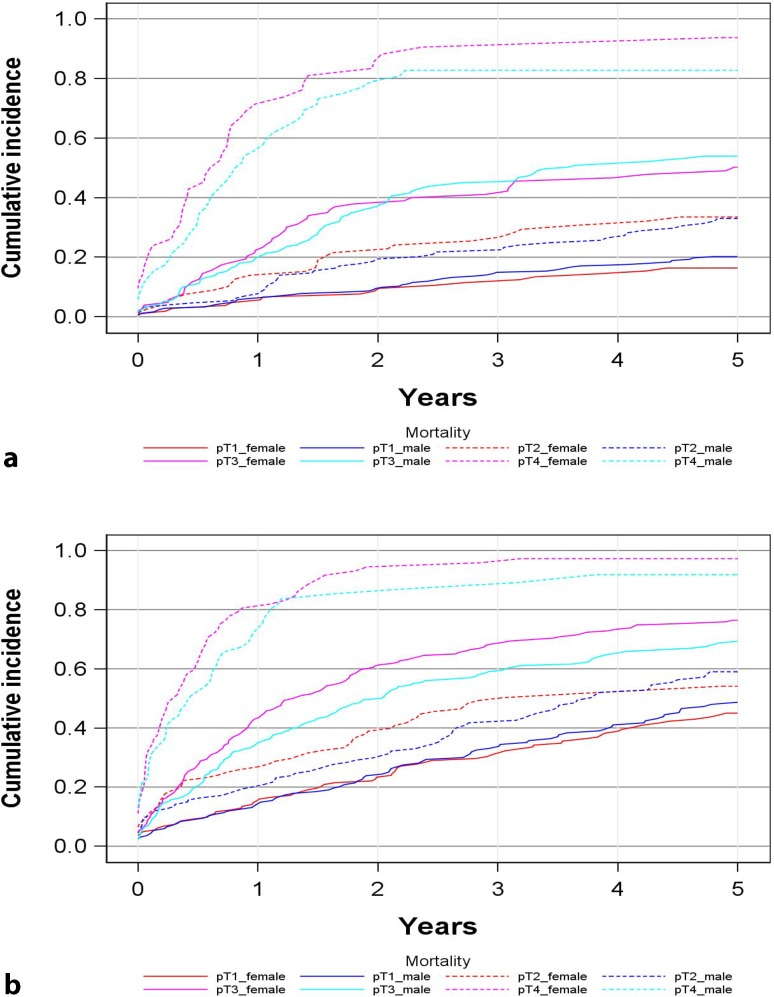
Cumulative overall mortality depending on sex and tumor stage in two age cohorts (**a** <70 years at diagnosis, **b** ≥70 years at diagnosis)

The 5‑year cumulative overall mortality was 57 % (95%CI: 53–59 %) for women versus 50 % (47–53 %) for men (*p* = 0.0002) (Fig. 1, Table 2). For pT1 tumors women 33 % (27–39 %), men 31 % (27–36 %) and pT2 tumors women 45 % (38–52 %), men 45 % (39–51 %) the 5‑year overall mortality rates were comparable (Fig. 2). In pT3 and pT4-tumors, however, women showed a higher cumulative mortality, in pT3 for women was 68 % (62–73 %), for men was 62 % (56–66 %) and for pT4 women 95 % (89–98 %) and men 87 % (79–92 %). To limit the impact of the age difference at diagnosis (see Table 1) we grouped patients into a <70 years (men 59.5 ± 8.5 years, women 61.5 ± 7.4 years) and a >70 years cohort (men 77.7 ± 5.2 years, women 78.9 ± 5.4 years) and performed a analysis by stage (Fig. 3). In principal, these data confirm those presented in Fig. 2.

**Table 2:** Overall mortality according to sex and tumor stage

Tumor stage	Alive (*n*)	Deceased (*n*)	Total
pT1 female	104	164	268
pT1 male	172	239	411
pT2 female	54	133	187
pT2 male	79	184	263
pT3 female	65	263	328
pT3 male	98	284	382
pT4 female	4	110	114
pT4 male	13	100	113
Total	589	1477	2066

Observed estimated differences between both sexes within tumor stages were not significant as assessed by a Cox and a Fine-Gray regression model for pT2 (*p* = 0.67) and pT3 tumors (*p* = 0.33). For pT4 tumors, the sex difference was significant (*p* = 0.044) and for pT1 tumors the difference was on the border of significance (*p* = 0.06).

### Cumulative cancer-specific mortality

**Fig. 4:**
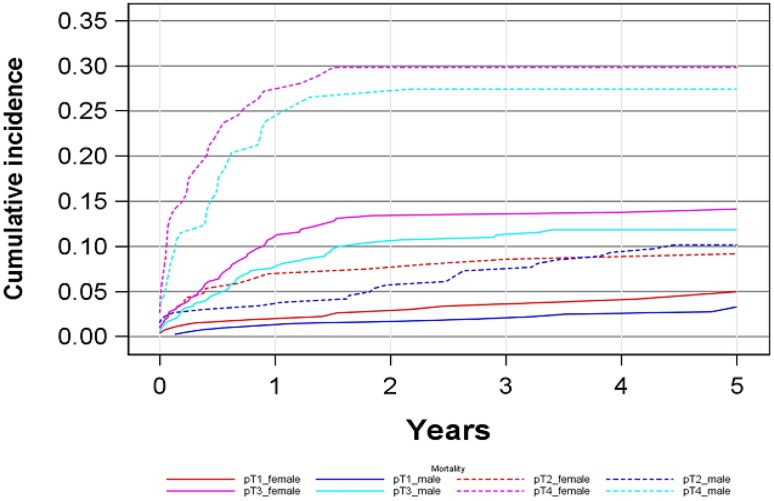
Cumulative cancer-specific mortality depending on sex and tumor stage

**Fig. 5:**
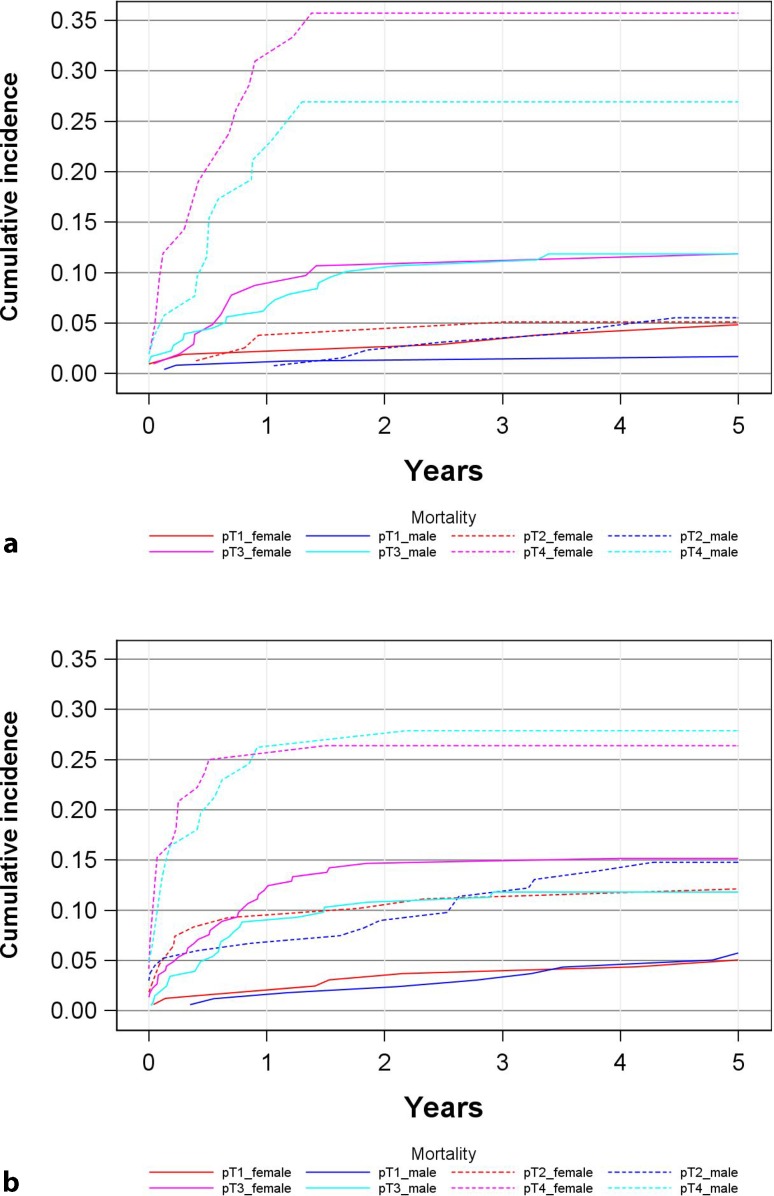
Cumulative cancer-specific mortality depending on sex and tumor stage in two age cohorts (**a** <70 years at diagnosis, **b** ≥70 years at diagnosis)

The 5‑year cancer-specific mortality (CSM) of the entire cohort was 12 % for women and 10 % for men (*p* = 0.067) (Fig. 4, Table 3). The 5‑year cumulative cancer and specific sex mortality was as follows: pT1 tumors 5 % (2–8 %) for women versus 3 % (1–5 %) for men, pT2 tumors 9 % (5–10 %) for women versus 10 % (6–10 %) for men, pT3 tumors 14 % (10–18 %) for women versus 11 % (8–15 %) for men and pT4 tumors 29 % (21–38 %) for women versus 27 % (19–35 %) for men. Fig. 5 presents CSM rates in the two age cohorts (<70 years vs. ≥70 years) thus largely confirming the data of the overall group (see Fig. 4). Observed estimated differences between groups within tumor stages were not significant as assessed by a Cox and a Fine-Gray regression model because of low numbers of events and subsequently low power of the statistical tests.

**Table 3:** Cancer-specific mortality according to tumor stage and sex

Tumor stage	Alive	Cancer deaths	Other deaths	Total
pT1 female	104	13	151	268
pT1 male	172	13	226	411
pT2 female	54	17	116	187
pT2 male	79	26	158	263
pT3 female	65	46	217	328
pT3 male	98	45	239	382
pT4 female	4	34	76	114
pT4 male	13	31	69	113
Total	589	225	1252	2066

## Discussion

This large-scale population-based analysis of UTUC has two major findings: (1) women presented with more advanced tumor stages and (2) women tended to have a higher overall and cancer-specific mortality in advanced tumor stages.

Prior discussion, some advantages and limitations of the current study design need to be mentioned. Strengths are (i) the population-based approach, (ii) the large cohort, (iii) the fact that for decades Austria has had an equal access healthcare system and (iv) that there is an almost complete follow-up (unless the patient left Austria) due to matching the cancer incidence database to the national mortality statistics by Statistic Austria. The major limitation is the lack of any clinical information including primary and secondary treatment; however, to be included to the Austrian Cancer registry a histological diagnosis is mandatory. It is the rare exception in Austria that a positive biopsy of UTUC is not followed by a nephroureterectomy (N/U); however, we have no information on the type of N/U, if a bladder cuff has been excised or the extent of lymphadenectomy. This population-based series with 2066 patients detected a fairly strong sex-specific tumor shift towards more advanced stages in women: the male to female ratio continuously declined from 1.5 for pT1-tumors to 1.4 for pT2 tumors, 1.2 for pT3 tumors and 1.0 for pT4 tumors. This trend, however, has not been uniformly observed. Shariat et al. [8], for instance, studied 754 patients (68.4 % men) undergoing N/U in a multicenter (*n* = 9) retrospective setting. In this series the male to female ratio increased from 1.8 for pT1 tumors to 2 for pT2 tumors and from 2.1 for pT3 tumors to 2.9 for pT4 tumors. In a further N/U series, Fernandez et al. [9] studied 1363 patients (67.6 %) men in a similar multicenter (*n* = 13) setting. The male to female ratio was comparable for all T stages (2.1 for pT1, 2.1 for pT2 and 2 for pT3). Further, smaller sized surgical series led to contradictory results in this respect [10–13]. Lughezzani et al. [14] studied 4850 patients (59.9 % men) who underwent N/U using the SEER data base. This large-scale population-based series partly supports our findings as the authors observed a higher proportion of women in pT3 stages (pT4 stages were not reported) [14]. The reasons for these discrepant data remain poorly understood. As pointed out by Lughezzani et al. [14], patient selection may be a factor as the majority of studies were surgical series from tertiary centers. Furthermore, many series are hampered by the limited sample size due to the low incidence of UTUC; however, the two largest, population-based studies in this respect support this gender-specific stage shift in UTUC. Our group has recently reported a similar phenomenon by analysing 27,733 patients with bladder cancer in Austria [15]. It has been postulated that a gender-specific referral pattern might be a major factor for the stage shift in women with bladder cancer [16] and one can speculate that the same also applies for UTUC. It is very likely that there is a lack of immediate diagnostic work-up of females with microhematuria. Other potential causes may be different carcinogen exposure and metabolism (e.g. tobacco and chemicals) as well as reflective of genetic, anatomical, hormonal and environmental factors. Similar to the situation for bladder cancer, these data should sensitize the medical community for immediate work-up of women, e. g. with hematuria.

The second part of the study deals with the impact of sex on overall and cancer-specific mortality; however, as in most cancer registry studies using gender as an independent predictor, there is the shortcoming that age at diagnosis was consistently more advanced in women (see Table 1). We have adjusted the effect of age in a descriptive and a statistical-inferential way. The statistical analyses, however, were hampered by the relatively low number of events particularly per stage group, when comparing the results, e. g. to a similar analysis of our group of bladder cancer patients using a 10-fold larger data base [15].

Similar to the issue of a gender-specific stage shift, the impact of female gender on the outcome of UTUC is a controversial matter. In our series, women had a higher overall (0.56 vs. 0.50, *p* = 0.0002) and cancer-specific mortality (0.12 vs. 0.10, *p* = 0.067). With respect to CSM our data are in line with the series of Lughezzani et al. [14] who also observed a higher CSM in women (*p* = 0.03). The 5‑year CSM rate in females was 16.9 % vs. 14.8 % in males in the SEER-database; these figures are remarkable similar to our series [14]. In stage-specific analyses, women had a higher CSM for pT2 and pT3 tumors [14]; however, in contrast to our series, men had a higher overall mortality in the advanced stages. Shariat et al. [8] reported on no gender difference regarding cancer-specific survival in a series of 754 patients who underwent N/U. Similar data were reported by Choo et al. [10]. These negative data are contrasted by Sikic et al. [11] who studied 268 patients and Liu et al. [12] who studied 285 patients. Both studies reported that female sex was an age-dependent prognostic factor for cancer specific survival for patients with UTUC treated with N/U.

## Conclusion

According to our knowledge, this is the largest population-based and nationwide series on the impact of gender on tumor stage and outcome of UTUC. Similar to the situation for bladder cancer, gender differences were notable for UTUC. Women tended to have more advanced tumor stages and a higher overall and cancer specific mortality in advanced stages. The statistical analyses were hampered by the rather low number of events particularly per stage group.
